# Analgesic Activity of 5-Acetamido-2-Hydroxy Benzoic Acid Derivatives and an In-Vivo and In-Silico Analysis of Their Target Interactions

**DOI:** 10.3390/ph16111584

**Published:** 2023-11-09

**Authors:** Cleydson B. R. Santos, Cleison C. Lobato, Sirlene S. B. Ota, Rai C. Silva, Renata C. V. S. Bittencourt, Jofre J. S. Freitas, Elenilze F. B. Ferreira, Marília B. Ferreira, Renata C. Silva, Anderson B. De Lima, Joaquín M. Campos, Rosivaldo S. Borges, José A. H. M. Bittencourt

**Affiliations:** 1 Laboratory of Modeling and Computational Chemistry, Department of Biological and Health Sciences, Federal University of Amapá, Macapá 68902-280, AP, Brazil; cleyson.cl@gmail.com (C.C.L.); camposchemistry@gmail.com (R.C.S.); recrisvale@hotmail.com (R.C.V.S.B.); maribrazaof@gmail.com (M.B.F.); 2 Graduate Program on Medicinal Chemistry and Molecular Modeling, Institute of Health Science, Federal University of Pará, Belém 66075-110, PA, Brazil; sayuriota@gmail.com (S.S.B.O.); rosborg@ufpa.br (R.S.B.); 3 Laboratory of Morphophysiology Applied to Health, State University of Pará, Belém 66095-662, PA, Brazil; jofre.freitas@uepa.br (J.J.S.F.); renatacs690@gmail.com (R.C.S.); andersonbentes@uepa.br (A.B.D.L.); 4 Laboratory of Organic Chemistry and Biochemistry, University of the State of Amapá, Macapá 68900-070, AP, Brazil; elenilze.batista@ueap.edu.br; 5 Department of Pharmaceutical and Organic Chemistry, Faculty of Pharmacy, Campus of Cartuja, University of Granada, 18071 Granada, Spain; jmcampos@ugr.es; 6 Biosanitary Institute of Granada (ibs.GRANADA), University of Granada, 18071 Granada, Spain

**Keywords:** analgesic, 5-acetamido-2-hydroxy benzoic acid, molecular docking, ADME, toxicity

## Abstract

The design, synthesis, and evaluation of novel non-steroidal anti-inflammatory drugs (NSAIDs) with better activity and lower side effects are big challenges today. In this work, two 5-acetamido-2-hydroxy benzoic acid derivatives were proposed, increasing the alkyl position (methyl) in an acetamide moiety, and synthesized, and their structural elucidation was performed using ^1^H NMR and ^13^C NMR. The changes in methyl in larger groups such as phenyl and benzyl aim to increase their selectivity over cyclooxygenase 2 (COX-2). These 5-acetamido-2-hydroxy benzoic acid derivatives were prepared using classic methods of acylation reactions with anhydride or acyl chloride. Pharmacokinetics and toxicological properties were predicted using computational tools, and their binding affinity (kcal/mol) with COX-2 receptors (*Mus musculus* and *Homo sapiens*) was analyzed using docking studies (PDB ID 4PH9, 5KIR, 1PXX and 5F1A). An in-silico study showed that 5-acetamido-2-hydroxy benzoic acid derivates have a better bioavailability and binding affinity with the COX-2 receptor, and in-vivo anti-nociceptive activity was investigated by means of a writhing test induced by acetic acid and a hot plate. PS3, at doses of 20 and 50 mg/kg, reduced painful activity by 74% and 75%, respectively, when compared to the control group (20 mg/kg). Regarding the anti-nociceptive activity, the benzyl showed reductions in painful activity when compared to acetaminophen and 5-acetamido-2-hydroxy benzoic acid. However, the proposed derivatives are potentially more active than 5-acetamido-2-hydroxy benzoic acid and they support the design of novel and safer derivative candidates. Consequently, more studies need to be conducted to evaluate the different pharmacological actions, the toxicity of possible metabolites that can be generated, and their potential use in inflammation and pain therapy.

## 1. Introduction

The inflammatory process has a great impact on several pathophysiological functions in living organisms. However, some events, especially those promoted by the uncontrolled immune system, have a negative effect on the body, such as inflammation, pain, aggression, and loss of function. One way to alleviate the symptoms resulting from this process is using non-steroidal anti-inflammatory drugs. However, these medications may have adverse effects such as gastric irritation, hepatotoxicity, nephrotoxicity, and other side effects [1,2]. The undesirable side effects promoted by these therapeutic agents have directed the search for new compounds for which the anti-inflammatory potential is accompanied by greater selectivity, greater specificity, minimal side effects and a low cost of production [3,4].

**Figure 1:**
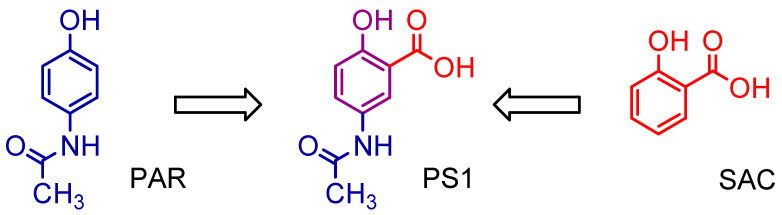
Chemical structure of 5-acetamido-2-hydroxy benzoic acid (PS1).

Moreover, 5-acetamido-2-hydroxy benzoic acid (PS1) was developed through rational drug design and molecular modeling using the molecular association between paracetamol (PAR) or acetaminophen and salicylic acid (SAC) at the Nucleus of Studies and Selection of Bioactive Molecules (NESBio) at the Federal University of Pará (UFPA, Brazil), in partnership with the research group Applied Computational Chemistry at the Federal University of Amapá (UNIFAP, Brazil—http://dgp.cnpq.br/dgp/espelhogrupo/10685, accessed on 10 March 2021) (see Figure 1). It showed a superior capacity when compared to other salicylates used in anti-inflammatory therapy and could represent an important strategy in the development of aspirin derivatives when applied to patients with a risk of resistance to the preventive treatment of cerebral vascular accident [5,6]. After that, a study to evaluate its acute oral toxicity, anti-nociceptive and anti-inflammatory properties was conducted by the pharmacology group at the College of Pharmacy at UFPA, Brazil [7,8].

Acute toxicity was based on OECD guideline 423, where no death was observed due to the administration of 5-acetamido-2-hydroxy benzoic acid at doses of 2000 and 5000 mg/kg. When comparing with their relatives, we emphasize a value of 1500 mg/kg for the acute toxicity (LD_50_) of acetylsalicylic acid (ASA) and 1944 mg/kg for acetaminophen (ACP) [6]. Next, the anti-nociceptive properties of 5-acetamido-2-hydroxy benzoic acid were investigated using the contortion, hot-plate and formalin tests in Swiss mice. The anti-edematogenic properties were evaluated using the carrageenan-induced paw edema model and croton oil-induced dermatitis in Wistar rats, and 5-acetamido-2-hydroxy benzoic acid did not promote behavioral changes or animal deaths during the acute evaluation of oral toxicity [6].

The 5-acetamido-2-hydroxy benzoic acid exhibited peripheric anti-nociceptive activity, evidenced by the reduction in the behavior of abdominal writhing induced by acetic acid, presenting an ED_50_ value of 4.95 mg/kg, as well as an anti-inflammatory effect in the formalin test in the inflammatory phase but not in the neurogenic phase [6]. When comparing this analgesic effect with its AAS relatives, which present values of 67.5 mg/kg and 125 mg/kg for the ACP, it shows a superior activity 10 to 25 times more potent than its precursors. In addition, 5-acetamido-2-hydroxy benzoic acid induced central anti-nociceptive activity in the hot-plate test both in the neurogenic phase and in the inflammatory phase of the formalin test, being effective in reducing edema formation induced by carrageenan or croton oil [6,7].

**Figure 2:**
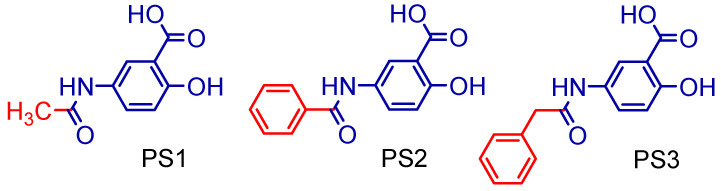
The 5-acetamido-2-hydroxy benzoic acid (PS1) and derivatives (PS2 and PS3).

The main problem with its possible therapeutic application was its low plasma bioavailability; the compound shows a peak in the first two hours and then begins to fall rapidly [7,8,9,10]. However, the changes in methyl red (PS1) in larger groups such as phenyl (PS2) and benzyl (PS3) aim to increase their selectivity over cyclooxygenase 2 (COX-2), also increasing the bioavailability parameters (see Figure 2).

In this study, the main objective was to evaluate the pharmacokinetic, toxicity, biological activity, and analgesia properties of the new 5-acetamido-2-hydroxy benzoic acid derivatives through the association of computational techniques and in-vivo experimental studies.

## 2. Results

### 2.1. Synthesis of 5-Acetamido-2-Hydroxy Benzoic Acid and Derivatives

**Figure 3:**
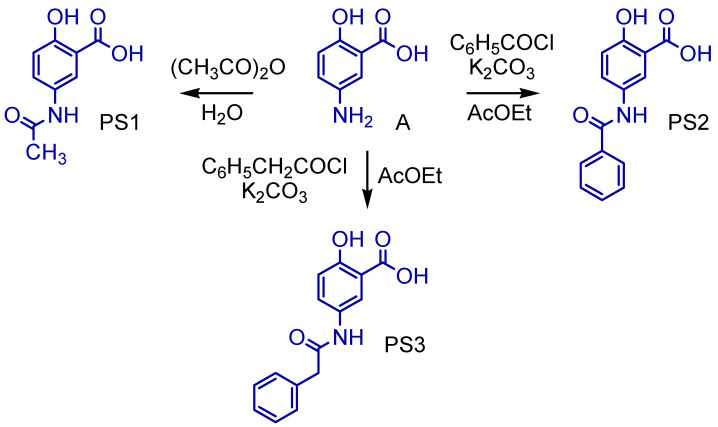
Synthetic methodology for the preparation of 5-acetamido-2-hydroxy benzoic acid and derivatives.

The 5-amino-2-hydroxy benzoic acid (A) was used as the starting reagent to obtain 5-acetamido-2-hydroxy benzoic acid and its derivatives by acetylation (PS1) and *N*-acylation reactions with benzoyl chloride (PS2) or phenylacetyl chloride (PS3), using water or ethyl acetate as solvents and potassium carbonate as a catalyst (see Figure 3).

The salicylamide (5-acetamido-2-hydroxy benzoic acid, PS1) was obtained by a reaction between 5-amino salicylic acid and acetic anhydride using water as a solvent. It was cold-soluble in ethanol, methanol, dimethylsulfoxide (DMSO), acetonitrile, acetic acid and acetone, and hot-soluble in ethyl acetate and water. The 5-benzamidosalicylic acid compound (PS2) was obtained by a reaction between 5-amino-salicylic acid and benzoyl chloride using ethyl acetate as a solvent and potassium carbonate as a catalyst. It was cold-soluble in methanol and DMSO and hot-soluble in ethyl acetate, acetonitrile, acetone, acetic acid and ethanol. The 5-phenylacetamidosalicylic acid compound (PS3) was obtained by a reaction between 5-amino-salicylic acid and phenylacetoyl chloride using ethyl acetate as a solvent and potassium carbonate as a catalyst. It was cold-soluble in methanol, DMSO, ethyl acetate, acetonitrile, acetone, acetic acid and ethanol.

### 2.2. In-Silico Study of Oral Bioavailability, Bioactivity, ADME and Toxicity Risk Assessment

Molecular weight is an important aspect of therapeutic drug action; if it increases beyond a certain limit, the bulkiness of the compounds also increases correspondingly, which in turn affects the drug action [11,12,13].

Even some drug molecules with a molecular weight higher than that established by Lipinski’s five rule (<500 Da) violate this principle and may have a good liposolubility profile. Druglikeness molecular descriptors of 5-acetamido-2-hydroxy benzoic acid derivatives are given in Table 1 and were tested against Lipinski’s rule of five. Compounds PS1, PS2 and PS3 have a molecular weight that varied from 208.21 to 271.27 Da, respectively. Thus, drug molecules that present MW < 500 are easily transported, diffused and absorbed as compared to heavy molecules [12] (see Table 1).

The activity of 5-acetamido-2-hydroxy benzoic acid derivatives against the GPCR ligand, ion channel modulator, kinase inhibitor, nuclear receptor ligand, protease inhibitor and enzyme inhibitory activity were predicted via the Molinspiration server (https://www.molinspiration.com, accessed on 21 November 2021.) and are summarized in Table 2. The molecules with a bioactivity score of more than 0.00 are likely to possess considerable biological activities, according to Roy et al. (2015) [13]. The values from −0.50 to 0.00 are expected to be moderately active, and if the score is less than −0.50, it is presumed to be inactive.

The prediction of Absorption, Distribution, Metabolism and Excretion (ADME) proprieties for 5-acetamido-2-hydroxy benzoic acid derivatives are shown in Table 3. Such ADME predictions were based on literature studies [14,15,16,17,18].

Table 4 shows the results of toxicity predictions using the identification of toxicophoric groups of 5-acetamido-2-hydroxy benzoic acid derivatives. This evaluation was conducted using the Deductive Estimation of Risk from Existing Knowledge (DEREK) 10.0.2 program [19,20,21]. We have considered DEREK alerts of toxicity involving the human species that are classified as plausible in mammals.

### 2.3. Molecular Docking Simulations

**Figure 4:**
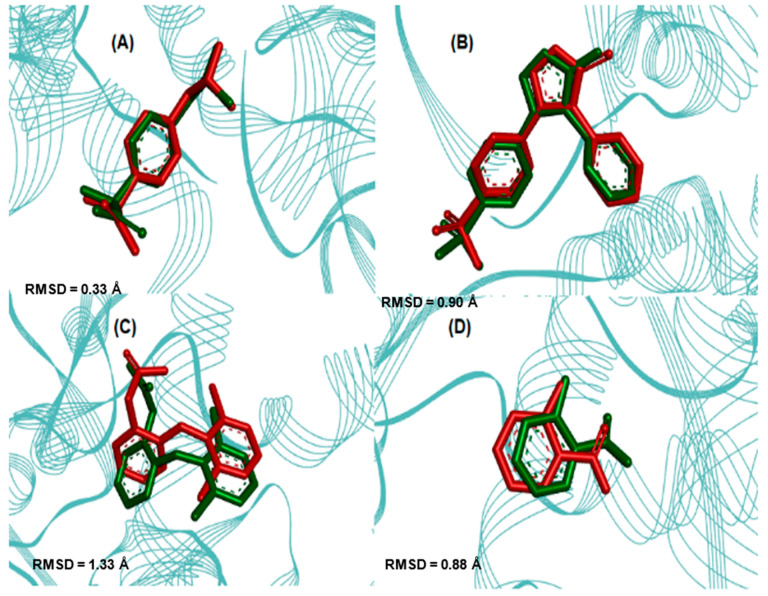
Superpositions of crystallographic ligands poses (in red) with the calculated poses (in green): (**A**) COX-2 (*Mus musculus*, PDB ID 4PH9–RMSD 0.33 Å), (**B**) COX-2 (*Homo sapiens*, PDB ID 5KIR–RMSD 0.90 Å), (**C**) COX-2 (*Mus musculus*, PDB ID 1PXX–RMSD 1.34 Å) and (**D**) COX-2 (*Homo sapiens*, PDB ID 5F1A–RMSD 0.88 Å) with respective crystallographic ligands.

The comparison between crystallographic ligands ibuprofen, rofecoxib, diclofenac and salicylic acid (red color) and the best conformation predicted by molecular docking (green color) can be seen in Figure 4, and the comparisons between experimental and predicted binding affinities are shown in Table 5. 

**Figure 5:**
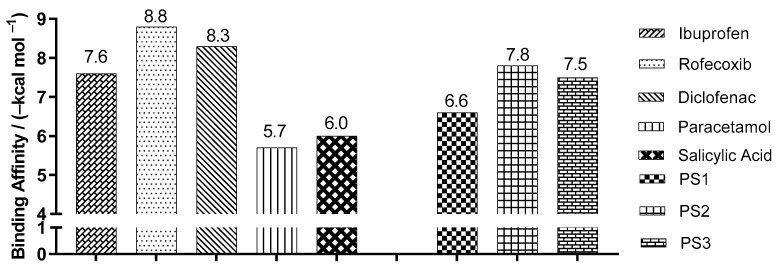
Binding affinity values of the ligands with COX-2 (*Mus musculus*) PDB ID 4PH9, by molecular docking.

The validation was accepted despite the minor deviation observed between the poses because the two crystallographic poses are possible. This was in order to evaluate whether the changes made would lead to a higher binding affinity than the respective inhibitor to each target. After validation, molecular docking was used to predict the modes of interaction and binding affinity of the PS1, PS2 and PS3 ligands. Figure 5 shows the best binding affinity values, in kcal/mol, of the ligands with COX-2 (PDB ID 4PH9).

**Figure 6:**
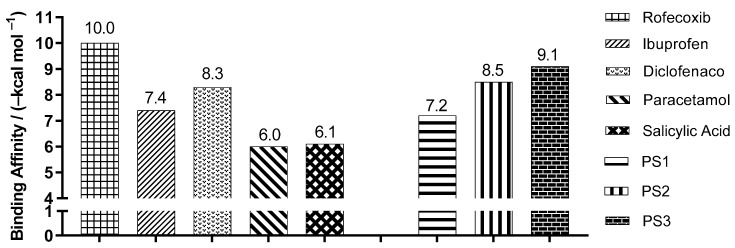
Binding affinity values of the ligands with COX-2 (*Homo sapiens*) PDB ID 5KIR, obtained by molecular docking.

Figure 6 shows the best binding affinity values, in kcal/mol, of the ligands with COX-2 (PDB ID 5KIR) of *Homo sapiens*. The control structure of rofecoxib exhibited a binding affinity of −10.0 kcal/mol, lower than PS1, PS2 and PS3, which presented values of −7.2, −8.5, −9.1 kcal/mol, with a variation of ±2.8, ±1.5 and ±0.9 kcal/mol, respectively.

**Figure 7:**
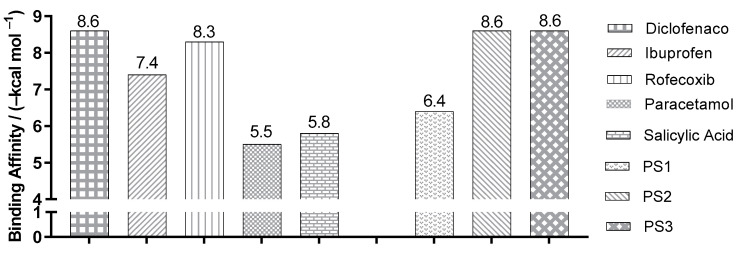
Binding affinity values of the ligands with COX-2 (*Mus musculus*) PDB ID 1PXX, obtained by molecular docking.

Figure 7 shows the best binding affinity values, in kcal/mol, of the ligands with COX-2 (PDB ID 1PXX) of *Mus musculus.* The control structure of diclofenac exhibited a binding affinity of −8.6 kcal/mol, smaller than PS1, varying by ±2.2 kcal/mol. When compared to the PS2 and PS3 structures, the affinity values were the same.

**Figure 8:**
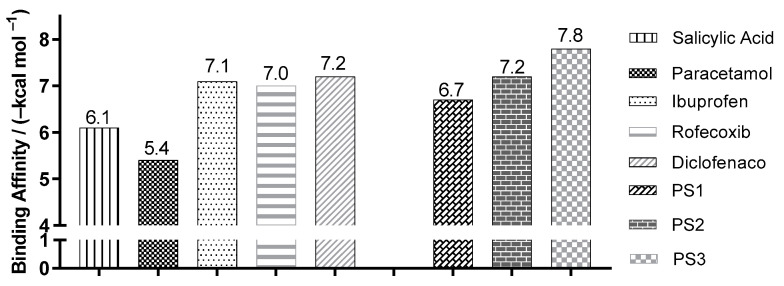
Binding affinity values of the ligands with COX-2 (Homo sapiens) PDB ID 5F1A, obtained by molecular docking.

Figure 8 shows the best binding affinity values, in kcal/mol, of the ligands with COX-2 (PDB ID 5F1A) of *Homo sapiens*. The control structure of salicylic acid exhibited a binding affinity of −6.1 kcal/mol. Compared with salicylic acid, PS1, PS2 and PS3 showed better bond affinity values of −6.7, −7.2 and −7.8 kcal/mol, with a variation of ±0.6, ±1.1 and ±1.7 kcal/mol, respectively.

**Figure 9:**
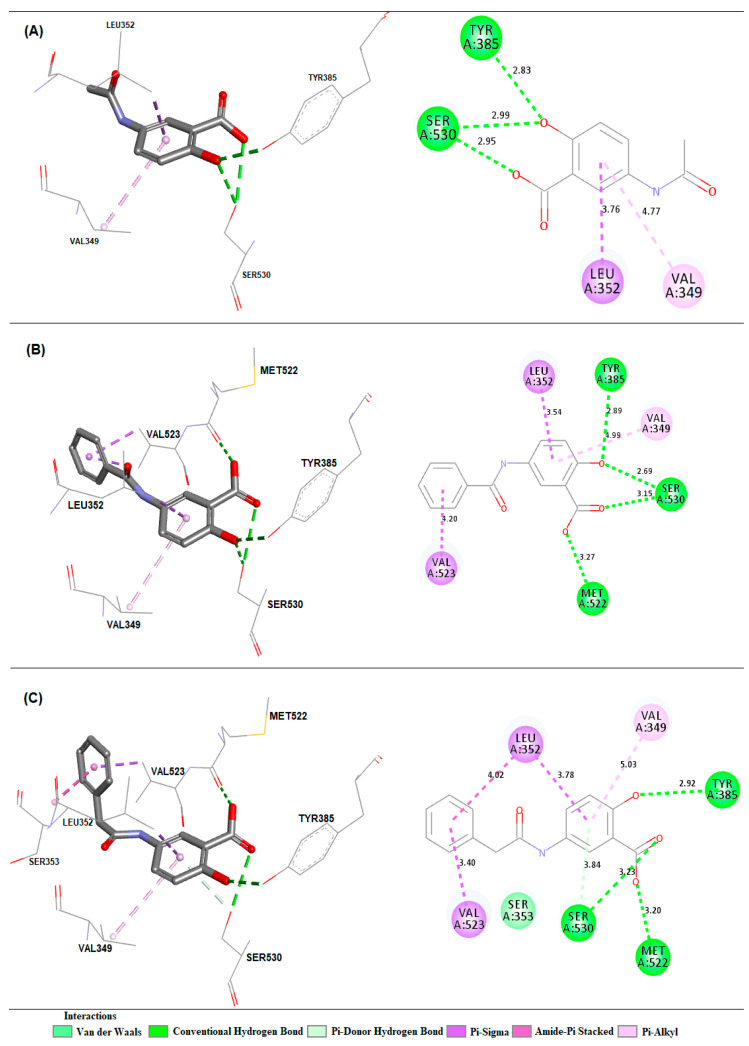
Interactions and distances (Å) predicted between the active site of COX-2 and compound PS1 (**A**), PS2 (**B**) and PS3 (**C**).

Crystallographic structures of the COX-2 enzyme with carboxylic acid-containing non-steroidal anti-inflammatory drugs show that the inhibitors are positioned in a similar manner with their coordinated carboxylates for Arg120 and their aromatic functionality protruding into the active site of cyclooxygenase toward Tyr385 [24] (see Figure 9).

### 2.4. Anti-Nociceptive Activity

#### 2.4.1. Central Analgesic Activity

**Figure 10:**
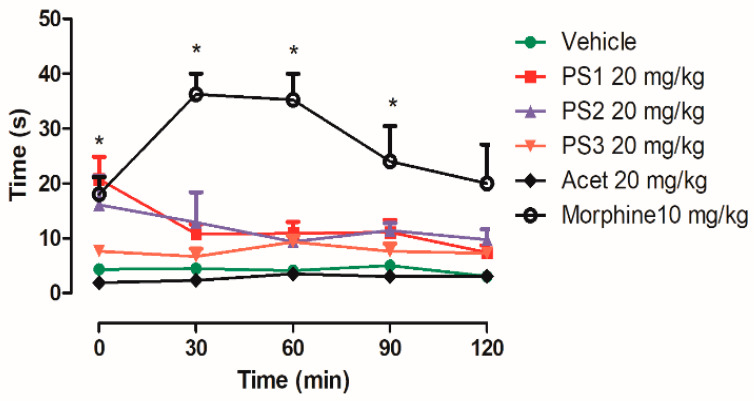
Effect of PS1, PS2, PS3 and Acetaminophen (Acet) at a dose of 20 mg/kg and morphine at a dose of 10 mg/kg on the nociceptive heat stimulus (55 ± 0.1 °C) induced in mice. Each point represents mean ± e.p.m. of five animals. * *p* < 0.05, when compared to the control group; ANOVA, Dunn’s method.

PS1 at a dose of 20 mg/kg prolonged the increase in latency (the time that the animals needed to manifest a stereotyped reaction to the heat stimulus) in a significant way in 30 min, and PS2 had a high variation in the same time period. PS3 and acetaminophen did not show central analgesic activity when compared to the control group. However, morphine (an opioid drug) significantly prolonged the latency at 30, 60 and 90 min in the hot-plate test (see Figure 10).

#### 2.4.2. Peripheral Analgesic Activity

**Figure 11:**
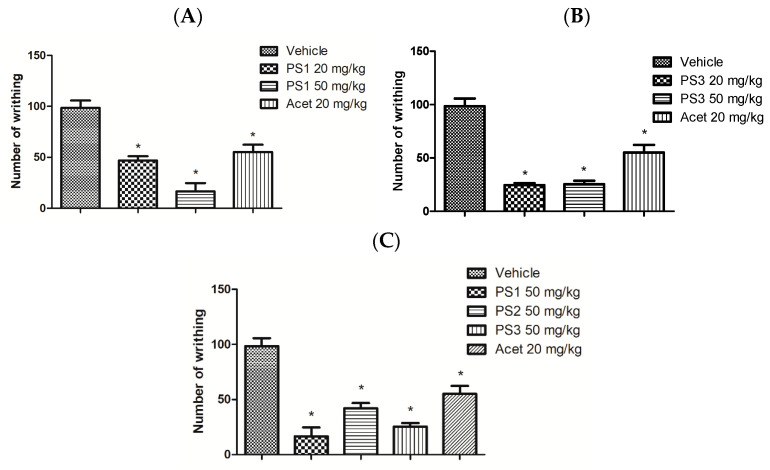
(**A**) Effect of PS1 and Acetaminophen (Acet) on the nociceptive stimulus induced by intraperitoneal injection of 0.6% acetic acid in mice. (**B**) Effect of PS3 and Acet on the nociceptive stimulus induced by the intraperitoneal injection of 0.6% acetic acid in mice. (**C**) Comparison of compounds (PS1, PS2 and PS3 at a dose of 50 mg/kg) with Acet at a dose of 20 mg/kg on the nociceptive stimulus induced by the intraperitoneal injection of 0.6% acetic acid in mice. Each column represents mean ± e.p.m. of five animals. * *p* < 0.05, when compared to the control group; ANOVA, Student-Newman–Keuls test.

In this model, acetic acid releases endogenous mediators (histamine, serotonin, bradykinin, substance P, and prostaglandins, especially PGI2, as well as some cytokines such as interleukin 1β (IL-1β), tumor necrosis factor (TNF-α) and interleukin 8) that are involved in the development of inflammatory pain. Acetaminophen is an analgesic drug used as the standard drug in this study (control group) (see Figure 11).

## 3. Discussion

### 3.1. Synthesis of 5-Acetamido-2-Hydroxy Benzoic Acid and Derivatives

Compound PS1 is insoluble in dichloromethane, chloroform and hexane. It is a colorless crystalline solid and has a melting point between 228.0 and 230.0 °C [28]. ^1^H NMR (300 MHz, DMSO-d6) δ (ppm) 6.89 (1H, d, J = 9.0 Hz, H-3), 7.64 (1H, dd, 9.3 and 2.7 Hz, H-4), 8.08 (1H, d, J = 2.7 Hz, H6), 2.00 (3H, s, CH_3_, C-8), 9.87 (1H, s, NH). ^13^C NMR (75 MHz; DMSO-d6) δ (ppm) 112.52 (C-1), 157.16 (C-2), 117.30 (C-3), 120.54 (C-4), 131.28 (C-5), 127.54 (C-6), 171.97 (COOH, C-7), 168.22 (CONH, C-8), 23.94 (CH_3_, C-9).

**Figure 12:**
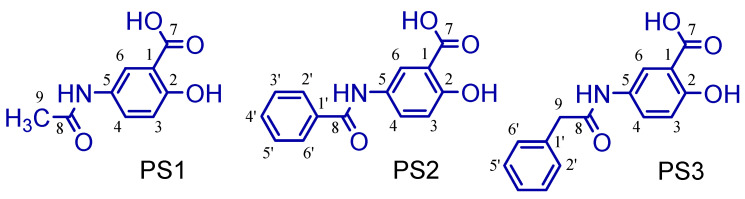
Structure and numeration of all derivatives studied here (PS1, PS2 and PS3).

The 5-benzamidosalicylic acid compound, PS2, is insoluble in dichloromethane, chloroform and hexane. It is a crystalline solid of slightly white coloration and has a melting point between 278.4 and 279.8 °C [28]. ^1^H-NMR (300 MHz; DMSO-d6) δ (ppm) 6.97 (1H, d, 9.0 Hz, H-3), 7.89 (1H, dd, 8.7 and 2.7 Hz, H-4), 8.29 (1H, d, 2.7 Hz, H-6), 10.24 (1H, NH), 7.96 (2H, dd, 6.9 and 1.2 Hz, H-2′ and H-6′), 7.53 (3H, m, H-3′, H-4′ and H-5′). ^13^C NMR (75 MHz; DMSO-d6) δ (ppm) 112.54 (C-1), 157.56 (C-2), 117.21 (C-3), 130.99 (C-4), 131.66 (C-5), 128.80 (C-6), 171.92 (COOH, C-7), 165.39 (CONH, C-8), 121.97 (C-1′), 127.71 (C-2′ and C-6′), 128.52 (C-3′ and C-5′), 134.87 (C-4′). All compounds and the numeration studied here are shown in Figure 12.

The 5-phenylacetamidosalicylic acid compound, PS3, is insoluble in dichloromethane, chloroform and hexane. It is a crystalline solid of white coloration with a melting point between 296.4 and 298.8 °C. ^1^H-NMR (300 MHz; CD_3_COCD_3_) δ (ppm) 6.90 (1H, d, 9.0 Hz, H-3), 7.75 (1H, dd, 8.7 and 2.7 Hz, H-4), 8.27 (1H, d, 2.7 Hz, H-6), 9.27 (1H, sb, NH), 3.68 (2H, s, CH_2_, C-9), 7.33 (2H, dd, 6.9 and 1.2 Hz, H-2′ and H-6′), 7.40 (2H, m, H-3′ and H-5′), 7.26 (1H, m, H-4′). 13C NMR (75 MHz; CD_3_COCD_3_) δ (ppm) 117.11 (C-1), 158.06 (C-2), 111.72 (C-3), 131.16 (C-4), 135.99 (C-5), 129.32 (C-6), 171.44 (COOH, C-7), 168.69 (CONH, C-8), 39.40 (CH_2_, C-9), 128.25 (C-1′), 126.55 (C-2′ and C-6′), 127.88 (C-3′ and C-5′), 120.76 (C-4′). The compound was compared with previously published results [28].

### 3.2. In-Silico Study of Oral Bioavailability, Bioactivity, ADME and Toxicity Risk Assessment

In the process of the discovery and development of new drugs, the success rate of new candidates selected for clinical development is approximately 20% [29], with most of the difficulties attributed to nonviable pharmacokinetic properties. Properties include low absorption rate, high liver extraction, and hepatic clearance, which cause low and variable bioavailability. In-vitro metabolic studies provide guidance for subsequent clinical studies [11].

PS1 showed moderate activity as an ion-channel modulator and enzyme inhibitor. Otherwise, PS2 and PS3 showed moderate activity as GPCR ligands, ion-channel modulators, kinase inhibitors, nuclear receptor ligands, protease inhibitors and enzyme inhibitors, also presenting a better profile of biological activity than salicylate and acetaminophen.

Since the G-protein coupled receptors (GPCRs) represent the largest family of cell surface molecules involved in signal transduction [30], some ligands can bind and activate GPCRs, including light-sensitive compounds, odors, pheromones, hormones, neurotransmitters, and peptides or proteins [31,32,33,34].

Also, several GPCRs have been reported to be involved in the activation and regulation of the NLRP3 inflammasome by sensing multiple ions, metabolites, neurotransmitters [35] and other therapeutic applications [36,37], including cancer, on similar molecules related to analgesic and anti-inflammatory drugs [38].

Otherwise, these properties can be related to promiscuity molecular prediction. In fact, drug promiscuity is defined as the property of a drug to act with multiple molecular targets and exhibit distinct pharmacological effects [39]. These properties are little explored but can be used in the discovery of new compounds by exploring molecular patterns and multi-target activity spaces [40].

However, no experimental evidence was observed for rofecoxib and diclofenac on GPCRs. Finally, similar compound predictions can be performed on the Molinspiration server and other online programs using druglikeness property determination [39].

Additionally, it is known that GPCRs play important roles in inflammation due to inflammatory cells (polymorphonuclear leukocytes (PMN), monocytes and macrophages) being able to express GPCRs. NSAIDS are effective anti-inflammatory and pain-relieving agents primarily because they block the synthesis of prostaglandins, which are lipid-derived autacoids that can interact with about nine prostanoid receptors that couple to several GPCR proteins [40,41,42].

In addition to the well-known activity in cyclooxygenase (COX) receptors, there is additional evidence that diclofenac probably activates GPCRs. Prostaglandins (PGs) collectively interact with prostanoid receptors that associate with a variety of G proteins and are responsible for several features of inflammation, including pain and edema [40,41]. Among the various arachidonic acid metabolites, PGE2 interacts with several GPCRs.

Evidence suggests that ATP-sensitive K+ channels are important in the anti-nociceptive action of non-steroidal anti-inflammatory drugs (NSAIDs) [43,44,45]. This result demonstrates the probable participation of the G+ channels coupled to the G protein in the internal rectifier and in the anti-nociceptive effect of diclofenac [43,44,45].

Thus, the prediction of the bioactivity of new drug candidates is critical for describing the possible mechanisms of action. In addition, it may assist in the selection of experimental tests to evaluate their pharmacological effects [46,47,48].

The prediction of human intestinal absorption is a major objective in the optimization and selection of candidates for the development of oral medications. The focus on the discovery of modern drugs is not simply on the pharmacological activity but is also on the search for more favorable pharmacokinetic properties [15].

The results of human intestinal absorption are the sum of absorption and bioavailability, evaluated from the proportion of excretion or cumulative excretion in urine, bile and feces [15,16]. The investigated compounds PS2 and PS3 showed, through prediction, good human intestinal absorption, with values of HIA >90%; this was not the case for PS1, which showed the lowest absorption of 75.9775%.

P_Caco-2_ (nm/s) and P_MDCK_ (nm/s) cell models have been used as reliable in-vitro models for the prediction of oral drug absorption, with the Caco-2 cells being derived from human colon adenocarcinoma, and they have various routes of drug transport through the intestinal epithelium [15,16].

The compounds in Table 3 show a mean permeability of 19.6422, as proposed by Yazdanian et al. (1998) [17]. PS2 showed the lowest value of cell permeability with 18.293 nm/s. According to Irvine et al. (1999) [18], an MDCK cell system can be used as a tool for the rapid screening of permeability. Only PS2 and PS3 showed high permeability in the cell system MDCK (>25). PS1 had the lowest permeability values of MDCK of 5.172 when compared to the controls, PS2 and PS3. The distribution of a drug depends on its plasma protein binding (PPB) and partition in adipose tissue and other tissues. In plasma, the drug may be in an unbound or a bound form, which depends on the affinity that the drug presents by the plasmatic protein (drug target). If the protein binding is reversible, then a chemical equilibrium will exist between bound and unbound states. The protein binding can influence the biological half-life in the body. The bound portion may act as a reservoir or deposit to which the drug is slowly released in the unbound form. As the non-bound form is metabolized and/or excreted from the body, the fraction it is bound to will be released in order to maintain that balance [49,50].

Table 3 shows the results of the distribution properties (PPB% and C_Brain_/C_blood_) for the investigated compounds. The salicylamide derivatives PS2 and PS3 showed moderate plasma protein binding with a PPB of 69.05416% and 66.41028%, respectively.

The penetration of the blood–brain barrier is critical in the pharmaceutical field because compounds that act on the central nervous system (CNS) should go through it and inactive compounds in the CNS should not, in order to avoid collateral effects of the CNS [51]. The investigated compounds (PS1, PS2 and PS3) do not show an absorption value in the CNS greater than 1, and this agrees with the classification proposed by Ma et al. (2005) [52]. Compounds that showed a value greater than 1 (C_Brain_/C_Blood_ > 1) were classified as active in the CNS and may cause collateral effects. Compounds that showed values below 1 (C_Brain_/C_Blood_ < 1) were classified as inactive in the CNS, as seen in Table 3.

The DEREK program makes the prediction of the toxicity of the compounds in a qualitative way; it is a specialist system that focuses attention on the toxic action of chemical compounds. The system performs this analysis based on implemented rules and depicts the relationship between a structural feature and toxicophoric groups present in the compounds, which are possible inducers of certain types of toxicity. It is considered that in addition to toxicity, DEREK can identify aspects related to carcinogenicity, mutagenicity, skin sensitization, irritation, teratogenicity and neurotoxicity [19,20,21].

The anti-inflammatory drugs derived from 2-arylacetic and 3-arylpropionic acids, such as ibuprofen and diclofenac, may cause hepatotoxicity and the irritation of the gastric mucosa. The hepatotoxicity is due to an idiosyncratic metabolic and/or immune reaction, not associated with the fact that mice treated with benoxaprofen developed this problem [53]. In studies carried out by Geneve et al. (1987) [53], high doses of pirprofen and ibuprofen were shown to significantly inhibit the mitochondrial beta-oxidation of fatty acids in mice, leading to the microvesicular steatosis of the liver. This may lead to less serious events such as mild elevations in serum transaminases to severe hepatocellular and/or cholestatic injury, which are relatively rare but important due to the potential for progression to fulminant hepatic failure. Jaundice, centrilobular necrosis, microvesicular steatosis, fibrosis, and symptoms suggestive of a hypersensitivity syndrome are clinical findings associated with the use of these agents [54,55].

The derivation of modified protein adducts is thought to be essential to the hepatotoxicity induced by these agents. Two alternative metabolic pathways may play a causative role: hepatic acyl glucuronidation catalyzed by the uridine diphospho-glucurosyl transferase (UGT) system and acyl-coenzyme A (acyl-CoA) formation. It is well established that acyl glucuronides are reactive electrophiles that can undergo covalent u with plasma or tissue proteins via a transacylation or glycation mechanism [53,55].

In the former, protein adducts are formed by the nucleophilic displacement of the glucuronic acid moiety. In the latter, intramolecular migration of the acyl residue allows for the opening of the glucuronic acid ring to create an aldehyde intermediate susceptible to nucleophilic attacks [53,55]. However, the gastric mucosal irritation of the nonsteroidal anti-inflammatory propionic acid (NSAIDs are mainly a consequence of the physicochemical disruption of the gastric mucosal barrier, resulting from alpha-substituted propionic acids or esters) causes a loss in protection resulting from the inhibition of the cyclo-oxygenase activity of the gastrointestinal mucosa [21]. Cyclo-oxygenase inhibition is also the mechanism by which propionic acids, and other NSAIDs, exert their anti-inflammatory action [56].

Diclofenac can cause distinct forms of nephrotoxicity in man and other mammals because of the presence of aryl or fulvenyl acetic or 2-propionic acid derivative, which have all been associated with a high incidence of dose-independent idiosyncratic acute renal failure (ARF) secondary to acute interstitial nephritis (AIN) in man, with or without nephrotic syndrome typically characterized by (membranous) glomerulonephritis or minimal change disease [53,54,55,56,57,58,59].

The presence of phenols substituted in acetaminophen in the para-position by nitrogen or oxygen gives a positive response in the in-vitro chromosome aberration test in human lymphocytes, as well as in the L5178Y TK+/− assay [58,59,60]. Activity is generally observed in both the presence and absence of the S9 mix. Since such compounds are not associated with significant activity in the Ames test, this suggests that the response in the L5178Y TK+/− assay is most likely due to a chromosomal rather than a mutagenic effect. Possible mechanisms for the observed activity include the generation of reactive oxygen species (ROS), metabolism to quinone-type intermediates or the inhibition of the enzyme ribonucleotide reductase [58,59,60] (see Table 4).

Acetaminophen and salicylamide derivatives (PS1, PS2 and PS3) showed toxicity alerts for hepatotoxicity, probably due to the presence of the toxicophoric group p-aminophenol and has been proven to require metabolic activation mediated by the cytochrome P450 system. The reactive metabolite is thought to be *N*-acetylbenzoquinone imine (NAPQI). After a toxic dose of the compound and subsequent glutathione depletion, NAPQI can covalently bind to a number of intracellular target proteins, resulting, in particular, in mitochondrial damage and ATP depletion. Other factors such as inflammatory cytokines, oxidative stress, tyrosine nitration, and mitochondrial permeability transition are also thought to play a causative role [61,62,63,64,65]. PS2 showed alerts for carcinogenicity in mice and rats and peroxisome proliferation in mice and rats, but these effects are not seen in higher mammals, including in man.

Depending on the toxicity, classes are defined according to the globally harmonized system of classification of labelling chemicals (GHS) [15]. Table 4 shows clearly that the LD_50_ prediction values were 2800 (PS1), 2400 (PS2) and 2175 (PS3) mg/kg. Thus, the compounds of the present study have an advantage over the commercial compound. This shows that such compounds may have greater safety in use, since they may be used in a higher concentration than the commercial compound. From the results, it was clear that the commercial compounds should be given with more caution under experimental study to avoid any loss of animals due to toxicity, which may, in turn, affect the statistical analysis of the experiment.

### 3.3. Molecular Docking Simulations

The comparison between crystallographic ligands ibuprofen, rofecoxib, diclofenac and salicylic acid and the best conformation predicted by molecular docking is seen in Figure 4, which shows the poses with RMSD values of 0.33, 0.90, 1.34 and 0.88 Å for ibuprofen, rofecoxib, diclofenac and salicylic acid, respectively. The docking had the ability to reproduce the experimental binding affinities, and according to the literature, the mode of connection prediction using docking should present an RMSD value <2.0 Å in the crystallographic pose of the binder [66,67,68,69,70]. So, the search parameters are suitable for the docking step. We have emphasized similar results in our previous work using molecular docking tools to search for new potential leads or hits [21,71,72,73,74].

We identified the sites of interaction described for rofecoxib (PDB ID 5KIR) around the alpha-helix located between the amino acid residues Thr118-Ser121, Tyr348-Leu352 and Met522-Gly526, as well as around the beta-sheet located between the amino acid residues Ser353-Phe357. For the binder, it is possible to observe common hydrogen bonds with residues Tyr355 and Arg120. There are also hydrophobic interactions with the residues Val349, Ser353, Leu352, Val523 and Ala527, according to studies in the literature [23].

In molecular docking, the theoretical binding affinity for ibuprofen was −7.6 kcal/mol, and a variation of ±0.3 kcal/mol was observed when compared with the experimental value of −7.3 kcal/mol [22]. For rofecoxib, the theoretical binding affinity was −10.0 kcal/mol, and a variation of ±0.8 kcal/mol was found when compared with the experimental value of −9.2 kcal/mol [23]. For diclofenac, the variation was ±2.9 kcal/mol, which was between the experimental bond affinity value of −11.3 kcal/mol [75] and the theoretical bond affinity for the docking of −6.1 kcal/mol. For salicylate, the binding affinity was −6.1 kcal/mol, varying by ±0.9 kcal/mol, when compared to the experimental value of −6.7 kcal/mol [25]. Thus, the molecular docking methodology was able to accurately reproduce the experimental binding modes for ibuprofen, rofecoxib and salicylic acid. However, the values obtained for diclofenac may be due to the low resolution of PDB 1PXX when compared to the other PDBs (see Table 5).

Using the docking methodology selected here, we identified a potential binding mode for the compound capable of interacting with the active site of COX-2, similar to the observed crystallographic pose for ibuprofen (PDB ID 4PH9) around the alpha-helix located between the amino acid residues Asn86-Thr93, Lys114-Ser121, Val349-Ser353, Met522-Lys532 as well as around the beta-sheet located between the amino acid residues Gly354-Phe357, Leu384-Trp387, Ala516-Gly519. For the binder, it is possible to observe common hydrogen bonds formed with residues Arg120 and Tyr355. There are also hydrophobic interactions with residues Val116, Val349, Trp387, Met522, Val523, Gly526, Ala527, Leu531 and Ser530, according to a study found in the literature [22].

For diclofenac (PDB ID 1PXX), the sites of interactions observed were around the alpha-helix located between the amino acid residues Met197-Phe205, Lys342-Ser353 and Val523-Met535, as well as around the beta-sheet located between the residues of amino acids Tyr385-His388. For the ligand, hydrogen bonds are observed with residues Tyr385 and Ser530, according to previously reported results [24].

The interactions observed for salicylic acid (PDB ID 5F1A) were around the alpha-helix located between the amino acid residues Tyr348-Leu352 and Met522-Ser530, as well as around the beta-sheet located between the amino acid residues Tyr385-His388. For the binder, a hydrogen bond with the Ser530 residue is observed, according to previous studies [25].

The control structure of ibuprofen showed a binding affinity of −7.6 kcal/mol, a close value when compared to PS1 and PS3, which presented values of −6.6 and −7.5 kcal/mol with variation of ±1.0 and ±0.1 kcal/mol, respectively. Compound PS2 presented a binding affinity value of −7.8 kcal/mol, a better value when compared to ibuprofen, with a variation of ±0.2 kcal/mol. When comparing the PS2 compounds with rofecoxib, a variation of ±1.0 kcal/mol was observed (see Figure 5).

The active site of the COX-2 enzyme has three important regions. First, there is the hydrophobic pocket, which is coated with the amino acid residues Tyr385, Trp387, Phe518, Ala201, Tyr248 and Leu352. The second region is located at the site entrance and contains the hydrophilic amino acid residues Arg120, Glu524 and Tyr355, while the third is a side pouch with the amino acid residues His90, Arg513 and Val523 [76]. Some amino acid residues (Arg120, Tyr355, His90, Arg513, Val523, Ser353 and Glu124) are believed to play a significant role in the entry of the ligand into the active site, as shown in the analysis of the crystal structure of various selective COX-2 inhibitors [77].

The individually observed interactions between PS1 and the active site are located around the alpha-helix between the amino acid residues Val349-Leu352 and Met522-Ser530, as well as around the beta-sheet located between the amino acid residues Tyr385-His388; they were similar to those described in the literature [22,24,76], as can be seen in Figure 9A. For PS1, hydrogen bonds formed with residues Ser530 and Tyr385 are observed. The latter residue participates in the catalytic process exerted by COX-2 [24,77] and hydrophobic interactions with Leu352 and Val349 residues. In the interactions observed between PS2 and PS3 compounds and the active site, both ligands exhibited the same hydrogen interactions with amino acid residues Ser530 and Tyr385 and hydrophobic interactions with residues Val349, Leu352, Met522 and Val523 (Figure 9B,C). Analyses in a crystallographic complex of COX-2 and arachidonic acid revealed a unique conformation of the substrate in which the substrate was inverted with its coordinated carboxylate for Ser530 and Tyr385 [23]. Studies by Rowlinson et al. (2003) [24] suggest that Tyr385/Ser530 chelation is critical for the inhibition of COX-2 by clinically used NSAIDs such as diclofenac, piroxicam and nimesulide.

### 3.4. Anti-Nociceptive Activity

The results presented in Figure 11A show the dose-dependent effect of PS1 on the number of abdominal writhes induced by 0.6% acetic acid. PS1 at doses of 20 and 50 mg/kg reduced the amount of abdominal writhing by 52% and 83%, respectively, when compared to the control group. In Figure 11B, the PS3, at doses of 20 and 50 mg/kg, reduced painful activity by 74% and 75%, respectively, when compared to the control group (20 mg/kg). PS2 in the dose of 50 mg/kg did not show good results when compared to the compounds (PS1 and PS3), and this can be seen in Figure 11C.

The anti-nociceptive potential of these salicylamide derivatives was evaluated using two different methodologies. The writhing test was used to evaluate the peripheral nociceptive activity in order to characterize the nature of the pain and indirectly verify the anti-inflammatory potential of the evaluated compounds. The hot-plate test was used to evaluate the involvement of the opioid system in the central nervous system.

## 4. Materials and Methods

### 4.1. Chemicals and Equipment

Varian Spectrometer, Gemini model: ^1^H-NMR (300 MHz) and ^13^C-NMR (75 MHz); Digital Fusion Point, model MQAPF–302 Microquímica (Campinas, Brazil). Analytical balance–Sartorios (Göttingen, Germany); Heating plate with magnetic stirrer–Quimis (Aguaí, Brazil); Becker (Carnegie, PA, USA) 100 mL and 250 mL; 125 mL Erlenmeyer, 250 mL and 500 mL; Petri dish; 25 mL, 50 mL and 100 mL beakers; 1 mL and 5 mL pipettes; 250 mL separation funnel; test tubes; watch glass; ice cube; glass rod; filter paper; chromatographic plate; magnetic bar; capillaries; Ethyl acetate–Synth (San Francisco, CA, USA); 5-Amino-salicylic acid–Fluka (Buchs, Switzerland); benzoyl chloride–Merck; phenylacetyl chloride–Aldrich (St. Louis, MO, USA); acetic acid–Nuclear (Diadema, Brazil); sulfuric acid–Synth; nitric acid–Synth; sodium chloride–New Chemistry (Campinas, Brazil); distilled water; acetonitrile–Merck (Rahway, NJ, USA); ethyl acetate; Nuclear; chloroform–Merck; dichloromethane-Synth; dimethylsulfoxide–Riedel-de-Haen (Berlin, Germany); ethanol Synth; hexane-Synth; methanol-Synth; toluene-Synth.

### 4.2. Synthetic Methodology

The acylation reactions were performed in temperature-controlled (60 to 80 °C) environments using an amine and anhydride or acyl chloride using a nucleophilic addition reaction of the amine group on acyl carbon followed by acetic anhydride or hydrochloride elimination. Under these reaction conditions, only the amine acylation occurs [11]. All derivatives were identified by means of physicochemical characterizations, such as melting points, solubility tests and H^1^ and C^13^ nuclear magnetic resonance (NMR) spectra [78,79,80,81].

### 4.3. In-Silico Study of Oral Bioavailability, Bioactivity, ADME and Toxicity Risk Assessment

The compounds used in this study were: ibuprofen, rofecoxib, diclofenac, acetaminophen, salicilate, PS1, PS2 and PS3. The molecular descriptors and druglikeliness (oral bioavailability) properties of the compounds were analyzed via the Molinspiration server (see site http://www.molinspiration.com, accessed on 21 November 2021) based on the Lipinski rule of five [82,83].

The rule states that most “druglike” compounds have a molecular weight (MW ≤ 500 Da), a number of hydrogen bond acceptors (HBA ≤ 10), a number of hydrogen bond donors (HBD ≤ 5), and an octanol/water partition coefficient (log P ≤ 5); compounds violating more than one of these rules can have problems with oral bioavailability. Molinspiration supports the calculation of important molecular properties such as MW, LogP, polar surface area, and the number of hydrogen bond donors and acceptors, as well as the prediction of the bioactivity score for the most important drug targets (GPCR ligands, kinase inhibitors, ion channel modulators, enzymes and nuclear receptors were predicted in this study [13]).

To identify any undesirable toxic properties of salicylamide derivates, the toxicity prediction server Protox [84] (see site http://tox.charite.de/tox, accessed on 11 January 2022) was used in this study. The prediction was based on the functional group similarity for the query compounds with the in-vitro and in-vivo validated compounds present in this database. The toxic properties such as toxicity class, toxic fragment generation, LD_50_ values in mg/kg, toxicity targets, drug-relevant properties [c Log P, Log S (Solubility)], molecular weight and overall drug score were calculated [84]. This approach was based on studies by Roy et al. (2015) [13].

The toxicity profile of the synthesized compounds was also evaluated using the Deductive Estimation of Risk from Existing Knowledge (DEREK) 10.0.2 program. We have considered DEREK alerts of toxicity involving the human species, mice and rats. The DEREK program conducts the prediction of the toxicity of the structures in a qualitative way and is a specialist system that focuses attention on the toxic action of chemical compounds. The system performs this analysis based on implemented rules and depicts the relationships between structural features and toxicophore groups present in the compounds as possible inducers of certain types of toxicity. It is considered that in addition to toxicity, DEREK can identify aspects related to carcinogenicity, mutagenicity, skin sensitization, irritation, teratogenicity and neurotoxicity [72,85,86].

### 4.4. Molecular Docking Simulations

The molecular docking study was performed with the aid of the AutoDock Vina 1.1.2 program [87] and its graphical interface PyRx 0.8 [88]. One of the most valuable features of docking methods is their ability to reproduce experimentally observed binding modes, even functioning as a form of validation. To perform a test of this level, a binder is extracted from its crystallographic complex and subjected to simulations with the binding site of the protein. Thus, the binding modes obtained in the simulations are compared with the respective binding modes obtained experimentally [87].

In this work, re-docking was performed to evaluate the accuracy of the docking program. The crystallographic models of cyclooxygenase-2 (COX-2) were selected from the Protein Data Bank (COX-2) and complexed with ibuprofen (PDB ID 4PH9) [22]; COX-2 (*Homo sapiens*) was complexed with rofecoxib (PDB ID 5KIR) [23]; COX-2 (*Mus musculus*) was complexed with diclofenac (PDB ID 1PXX) [24]; and COX-2 (*Homo sapiens*) was complexed with salicylic acid (PDB ID 5F1A) [25], with resolutions of 1.81, 2.69, 2.90 and 2.38 Å, respectively. 

The crystallographic structure obtained in the PDB was edited in the program Discovery Studio Visualizer v.17.2 [89] to separate the protein from the ligand, and then the two molecules were submitted to molecular docking in the program AutoDock Vina 1.1.2 [87]. The search space was defined using experimentally solved protein–ligand complex structures (PDBs) [22,23,24,25]. First, an initial docking box was constructed to enclose the bound ligand, and then the box size was increased until it included the entire region of the active site to allow for the rotation and translation of the ligand in this region.

Molecular docking was performed to obtain a population of possible conformations and orientations for the ligand at the binding site. The protein was loaded in PyRx, creating a PDBQT file that contains a protein structure with hydrogens in all polar residues. All calculations for protein-fixed ligand-flexible docking were performed using the Lamarckian Genetic Algorithm (LGA) method, which presents the best results in the search for the global minimum [90]. 

The docking site on the protein target was defined by establishing a grid box with the dimensions shown in Table 6. Ten runs via AutoDock Vina, with an exhaustiveness default = 8 [87], were performed in all cases per each ligand structure, and for each run the best pose with the lowest binding free energy and lower RMSD was selected, saved, and analyzed. The RMSD was calculated in the Discovery Studio Visualizer v.17.2 program by comparing the crystallographic ligand pose and the docking predicted pose using all atoms.

The X-ray diffraction (PDB ID 4PH9, 5KIR, 1PXX and 5F1A) was overlaid with the lowest RMSD conformation obtained in the docking to visually evaluate the best obtained result of the validation. After the identification with the best search parameters, the study of molecular docking with the PS1, PS2 and PS3 structures was carried out.

The X, Y and Z spatial coordinates were determined in the active site region according to the observed interaction between the enzymes and their respective crystallographic binders, according to studies available in the literature [91,92,93,94,95,96].

The coordinates used for the center and the box size can be seen in Table 6. Visualizations as well as distance measures of the interactions between the ligands and enzymes were produced using the program Discovery Studio Visualizer v.17.2.

### 4.5. Anti-Nociceptive Activity

This project is characterized as an experimental, prospective, and interventional study aimed at evaluating the in-vivo therapeutic efficacy of salicylamide (PS1), (PS2) and (PS3) derivatives in nociception models. This experimental study was carried out in the city of Belém-Pará, at UEPA’s Laboratory of Morphophysiology Applied to Health.

#### 4.5.1. Animals

This project is characterized as an experimental, prospective, and interventional study aimed at evaluating the in-vivo therapeutic efficacy of salicylamide (PS1) derivatives (PS2) and (PS3) in nociception models. All experiments reported in this study were conducted in accordance with current guidelines for care of laboratory animals and ethical guidelines for investigation of experimental pain in conscious animals (CEUA-UEPA Nº 14/2017).

Sixty-three Swiss mice belonging to the *Mus musculus* strain weighing between 30 g and 40 g were used. Adult males were used for each study group in vivo; they were from the Bioterio Evandro Chagas Institute (IEC) and were transferred and kept in the Luiz Carlos de Lima Silveira–UEPA/CCBS, where they were placed in appropriate acrylic cages with a bed of wood and kept at a temperature between 22 and 25 °C, receiving water ad libitum during the experiment period with a night light cycle/day of 12 h. The cleaning and the exchange of shavings and water were carried out on alternate days. The determination of the sample number was based on the central limit theory, once it was considered that the sample had a normal distribution.

#### 4.5.2. Writhing Test Induced by Acetic Acid

The writhing tests were induced by the intraperitoneal administration of 0.6% acetic acid. In this concentration, the contraction of the abdominal muscles is generated [97]. The animals were subdivided into five groups: acetaminophen, saline, PS1, PS2 and PS3, totaling 25 animals, and they were pre-treated orally. After 60 min, the acetic acid administration was performed. The anti-nociceptive activity was expressed by the number of contortions in 30 min.

#### 4.5.3. Hot Plate

The hot-plate test was used to measure the latency of response to the thermoceptive stimulus, according to the method described by Woolfe and Macdonald (1944) [98]. The mice were subdivided into six groups: vehicle, PS1, PS2, PS3, acetaminophen and morphine, totaling 30 animals. They were pre-treated orally with 20 mg/kg and only 10 mg/kg morphine. Each individual animal was placed on the hot plate (55 ± 0.5) °C. The time measurement was interrupted when the animal presented the instinctive behavior of jumping on the plate or licking or raising the legs, or after 35 s, to avoid tissue injury. The measurements to be analyzed were taken at the times of 30, 60, 90 and 120 min after the administration of the substance.

#### 4.5.4. Ethical Aspects

All animals in the present research were cared for according to the national legislation for Procedures for the Scientific Use of Animals in force (Federal Law 11,794 of 8 October 2008), the rules of the Brazilian College of Animal Experimentation (COBEA) (CEPA) of the University of the State of Pará (UEPA) and the coordinator of the Laboratory of Applied Morphophysiology for Health of UEPA/CCBS, and the guidelines and responsibilities for veterinarians.

## 5. Conclusions

Two salicylamide derivates were designed and their chemical structures were spectrally confirmed. The in-silico properties of pharmacokinetics and the bioactivity, bioavailability and toxicity of the compounds were analyzed. The compounds showed a better bioavailability, and the molecular docking results obtained here indicate that compounds PS2 and PS3 have a superior potential capacity of COX-2 inhibition in human and mice enzymes, as they exhibited similar interactions to those observed for the templates or control compounds (ibuprofen, diclofenac, acetaminophen, rofecoxib, and salicylate). In the anti-nociceptive activity, the compounds reduced the painful activity in different concentrations. However, the proposed derivatives are potentially more active than salicylamide and they support the design of novel and safer derivative candidates. Consequently, more studies need to be conducted to evaluate the different pharmacological actions, the toxicity of possible metabolites that can be generated, and potential uses in inflammation and pain therapy.

## Figures and Tables

**Table 1:** Oral bioavailability properties for 5-acetamido-2-hydroxy benzoic acid derivatives.

Compounds	MW ^1^ (<500 Da)	HBA ^2^ (≤10)	HBD ^3^ (≤5)	LogP (≤5) ^4^	MPSA (Å^2^) ^5^	MV (Å^3^) ^6^	NRB ^7^
Ibuprofen	206.28	2	1	3.46	37.30	211.19	4
Diclofenac	296.15	3	2	4.57	49.33	238.73	4
Acetaminophen	151.16	3	2	0.68	49.33	140.01	1
Salicylic acid	137.11	3	1	−1.81	60.36	116.32	1
Rofecoxib	314.36	4	0	0.71	60.45	264.79	3
PS1	208.21	5	3	1.06	86.62	167.01	2
PS2	210.19	5	3	2.74	86.62	221.86	3
PS3	271.27	5	3	2.83	86.62	238.66	4

^1^ Molecular weight, ^2^ Hydrogen bond acceptor, ^3^ Hydrogen bond donor, ^4^ Logarithm of the partition between of n-octanol and water phases, ^5^ MPSA (molecular polar surface area), ^6^ MV (molecular volume) and ^7^ NRB (number of rotatable bonds) were obtained.

**Table 2:** Bioactivity of the 5-acetamido-2-hydroxy benzoic acid derivatives and commercial drug ^1^.

Compounds	GPCR	Ion Channel Modulator	Kinase Inhibitor	Nuclear Receptor Ligand	Protease Inhibitor	Enzyme Inhibitor
Ibuprofen	−0.17	−0.01	−0.72	0.05	−0.21	0.12
Diclofenac	0.14	0.20	0.17	0.09	−0.10	0.25
Acetaminophen	−1.05	−0.54	−1.04	−1.21	−1.20	−0.68
Rofecoxib	0.20	−0.18	−0.18	0.12	0.26	0.61
Salicylic Acid	−1.00	−0.41	−1.26	−1.26	−1.13	−0.48
PS1	−0.67	−0.38	−0.70	−0.64	−0.76	−0.33
PS2	−0.20	−0.21	−0.13	−0.15	−0.25	−0.04
PS3	−0.10	−0.19	−0.15	−0.04	−0.14	−0.02

^1^ Score Values > 0.00 = considerable biological activities; Score Values −0.50 to 0.00 = moderately active; score values < −0.50 = considerable inactive.

**Table 3:** Absorption and distribution properties in percentages of PPB and penetration of the blood–brain barrier for 5-acetamido-2-hydroxy benzoic acid derivatives and commercial drug.

Compounds	Absorption			Distribuition	
	HIA ^1^	P_caco-2_ ^2^	P_MDCK_ ^3^	PPB (%) ^4^	C_Brain/_C_Blood_ ^5^
Ibuprofen	98.38	21.20	136.48	88.24	1.26
Diclofenac	95.95	24.53	51.46	91.95	1.39
Acetaminophen	88.23	18.78	15.43	0.00	0.61
Salicylic Acid	86.59	20.43	25.36	7.31	0.43
Rofecoxib	98.22	2.72	11.27	0.00	0.61
PS1	75.97	19.93	5.17	14.34	0.35
PS2	91.29	18.29	51.99	69.05	0.50
PS3	91.95	20.70	31.04	66.41	0.15

^1^ Percentage of human intestinal absorption; ^2^ cell permeability (Caco-2 in nm/s); ^3^ cell permeability Maden Darby Canine Kidney in nm/s; ^4^ percentage of plasma protein binding; ^5^ penetration of the blood–brain barrier.

**Table 4:** Toxicity prediction by the identification of toxicophoric groups and LD_50_ for 5-acetamido-2-hydroxy benzoic acid and derivatives.

Compounds	Toxicity Prediction Alert (Lhasa Prediction)	Toxicophoric Group	Toxicity Alert	LD_50_ Toxic ^1^	Toxicity Class ^2^
Ibuprofen	Hepatotoxicity in human, mouse and rat	2-arylacetic or 3-arylpropionic acid	PLAUSIBLE	299	III
		Alpha-substituted propionic acid or ester			
Diclofenac	Hepatotoxicity in human	2-arylacetic or 3-arylpropionic acid	CERTAIN	53	III
	Nephrotoxicity in human, mouse and rat	Aryl or fulvenyl acetic or 2-propionic acid derivative	PLAUSIBLE		
Acetaminophen	Chromosome damage in vitro in human	Phenol	CERTAIN	338	III
		Phenol	PROBABLE		
	Hepatotoxicity in human, mouse and rat	Para-aminophenol or derivative	CERTAIN		
Rofecoxib	-	-	NO ALERTS	4500	V
Salicylic Acid	-	-	NO ALERTS	480	IV
PS1	Hepatotoxicity in human, mouse and rat	Salicylic acid or analog	PLAUSIBLE	2800	V
		Para-Aminophenol or derivative			
PS2	Carcinogenicity in mouse and rat	Alkylaryl or bisaryl carboxylic acid or precursor	PLAUSIBLE	2400	V
	Hepatotoxicity in human, mouse and rat	Salicylic acid or analog			
	Peroxisome proliferation in mouse and rat	Para-aminophenol or derivative			
PS3	Hepatotoxicity in human, mouse and rat	Salicylic acid or analog	PLAUSIBLE	2175	V
		Para-aminophenol or derivative			

^1^ Values in mg/kg body weight. ^2^ Class I: fatal if swallowed (LD_50_ ≤ 5); Class II: fatal if swallowed (5 < LD_50_ ≤ 50); Class III: toxic if swallowed (50 < LD_50_ ≤ 300); Class IV: harmful if swallowed (300 < LD_50_ ≤ 2000); Class V: may be harmful if swallowed (2000 < LD_50_ ≤ 5000); Class VI: non-toxic (LD_50_ > 5000) [12].

**Table 5:** Comparison between experimental and theoretical binding affinities.

Enzyme COX2	Ligand	Experimental Binding Affinity * (kcal/mol)	Ki (nM)	Docking Predicted Binding Affinity (kcal/mol)	Resolution (Å)
(PDB ID 4PH9)	Ibuprofen	−7.3	7.2.10^3^ [22]	−7.6	1.81
(PDB ID 5KIR)	Rofecoxib	−9.2	310 [23]	−10.0	2.69
(PDB ID 1PXX)	Diclofenac	−11.3	1.10^4^ [24]	−8.6	2.90
(PDB ID 5F1A)	salicylic acid	−6.7	1.10^4^ [25]	−6.1	2.38
(PDB ID 4PH9)	Ibuprofen	−7.3	7.2.10^3^ [22]	−7.6	1.81

* Values calculated from experimentally determined inhibition constants (Ki), found in the PDBs, according to Equation: ΔG = R.T.lnKi [26,27], were R (constant of gas) = 1.987 · 10^−3^ kcal/(mol K) and T (temperature) = 310 K.

**Table 6:** Protocols used in the study of molecular docking study.

Enzyme COX2	Inhibitor *	Coordinates of the Grid Center (Angstrom)	Grid Dimensions (Angstrom)
(PDB ID 4PH9)	Ibuprofen	X = 12.58 Y = 24.20 Z = 25.33	X = 17 Y = 17 Z = 16
(PDB ID 5KIR)	Rofecoxib	X = 23.63 Y = 1.30 Z = 34.07	X = 18 Y = 19 Z = 18
(PDB ID 1PXX)	Diclofenac	X = 27.16 Y = 24.45 Z = 15.30	X = 18 Y = 19 Z = 18
(PDB ID 5F1A)	Salicylic acid	X = 41.72 Y = 24.24 Z = 240.03	X = 18 Y = 17 Z = 17

* Acetaminophen (Acet) and the crystallographic structures of ibuprofen, rofecoxib, diclofenac and salicylic acid were used as a positive control.

## Data Availability

Data is contained within the article.

## References

[1] Mansouri M.T., Hemmati A.A., Naghizadeh B., Mard S.A., Rezaie A., Ghorbanzadeh B. (2015). A study of the mechanisms underlying the anti-inflammatory effect of ellagic acid in carrageenan-induced paw edema in rats. Indian J. Pharmacol..

[2] Richy F., Bruyere O., Ethgen O., Rabenda V., Bouvenot G., Audran M., Herrero-Beaumont G., Moore A., Eliakim R., Haim M. (2004). Time dependent risk of gastrointestinal complications induced by non-steroidal anti-inflammatory drug use: A consensus statement using a meta-analytic approach. Ann. Rheum. Dis..

[3] Beck P.L., Xavier R., Lu N., Nanda N.N., Dinauer M., Podolsky D.K., Seed B. (2000). Mechanisms of NSAID-induced gastrointestinal injury defined using mutant mice. Gastroenterology.

[4] Chan F.K.L., Graham D.Y. (2004). Prevention of non-steroidal anti-inflammatory druggastrointestinal complications—Review and recommendations basedon risk assessment. Aliment. Pharmacol. Ther..

[5] Borges R.S. (2007). Planejamento, Síntese e Avaliação Antioxidante de Inibidores Fenólicos da PGES Derivados da Associação p-Aminofenol e Salicilatos.

[6] Borges R.S., Alves C.N., Nascimento J.L.M. (2010). Aplicação de Derivados da Associação Molecular como Antiagregantes Plaquetários e Inibidores de Radicais Livres.

[7] Borges R.S., Pereira G.A.N., Vale J.K.L., França L.C.S., Monteiro M.C., Alves C.N., Silva A.B.F.d. (2013). Design and evaluation of 4-aminophenol and salicylate derivatives as free-radical scavenger. Chem. Biol. Drug Des..

[8] Borges R.S., Castle S.L. (2015). The antioxidant properties of salicylate derivatives: A possible new mechanism of anti-inflammatory activity. Bioorganic Med. Chem. Lett..

[9] Guedes K.M.M., Borges R.S., Fontes-Júnior E.A., Silva A.S.B., Fernandes L.M.P., Cartágenes S.C., Pinto A.C.G., Silva M.L., Queiroz L.M.D., Vieira J.L.F. (2018). Salicytamide: A New Anti-inflammatory Designed Drug Candidate. Inflammation.

[10] Hawkey C.J. (2001). COX-1 and COX-2 inhibitors. Best Pract. Res. Clin. Gastroenterol..

[11] Tanhehco E.J. (2001). Potassium channel modulators as anti-inflammatory agents. Expert. Opin. Ther. Pat..

[12] Van De Waterbeemd H., Gifford E. (2003). ADMET in silico modelling: Towards prediction paradise?. Nat. Rev. Drug Discov..

[13] Roy S., Samant L., Chowdhary A. (2015). In silico pharmacokinetics analysis and ADMET of phytochemicals of Datura metel Linn. and Cynodon dactylon Linn. J. Chem. Pharm. Res..

[14] Cunha E.L., Santos C.F., Braga F.S., Costa J.S., Silva R.C., Favacho H.A., Hage-Melim L.I., Carvalho J.C., Silva C.H., Santos C.B. (2015). Computational Investigation of Antifungal Compounds Using Molecular Modeling and Prediction of ADME/Tox Properties. J. Comput. Theor. Nanosci..

[15] Yee S. (1997). In vitro permeability across caco-2 cells (colonic) can predict in vivo (small intestinal) absorption in man-fact or myth. Pharm. Res..

[16] Zhao Y.H., Le J., Abraham M.H., Hersey A., Eddershaw P.J., Luscombe C.N., Platts J.A. (2001). Evaluation of human intestinal absorption data and subsequent derivation of a quantitative structure activity relationship (QSAR) with the Abraham descriptors. J. Pharm. Sci..

[17] Yazdanian M., Glynn S.L., Wright J.L., Hawi A. (1998). Correlating Partitioning and Caco-2 Cell Permeability of Structurally Diverse Small Molecular Weight Compounds. Pharm. Res..

[18] Irvine J.D., Takahashi L., Lockhart K., Cheong J., Tolan J.W., Selick H.E., Grove J.R. (1999). MDCK (Madin–Darby canine kidney) cells: A tool for membrane permeability screening. J. Pharm. Sci..

[19] Costa J.S., Costa K.S.L., Cruz J.V., Ramos R.S., Silva L.B., Brasil D.S.B., Silva C.H.T.P., Santos C.B.R., Macêdo W.J.C. (2018). Virtual screening and statistical analysis in the design of new caffeine analogues molecules with potential epithelial anticancer activity. Curr. Pharm. Des..

[20] Cruz J.V., Neto M.F.A., Silva L.B., Ramos R., Costa J., Brasil D.S.B., Lobato C.C., Costa G.V., Bittencourt J.A.H.M., Silva C.H.T.P. (2018). Identification of novel protein kinase receptor type 2 inhibitors using pharmacophore and structure-based virtual screening. Molecules.

[21] Alberga D., Trisciuzzi D., Mansouri K., Mangiatordi G.F., Nicolotti O. (2018). Prediction of Acute Oral Systemic Toxicity Using a Multifingerprint Similarity Approach. Toxicol. Sci..

[22] Orlando B.J., Lucido M.J., Malkowski M.G. (2015). The structure of ibuprofen bound to cyclooxygenase-2. J. Struct. Biol..

[23] Orlando B.J., Malkowski M.G. (2016). Crystal structure of rofecoxib bound to human cyclooxygenase-2. Acta Crystallogr. Sect. F Struct. Biol. Commun..

[24] Rowlinson S.W., Kiefer J.R., Prusakiewicz J.J., Pawlitz J.L., Kozak K.R., Kalgutkar A.S., Stallings W.C., Kurumbail R.G., Marnett L.J. (2003). A novel mechanism of cyclooxygenase-2 inhibition involving interactions with Ser-530 and Tyr-385. J. Biol. Chem..

[25] Lucido M.J., Orlando B.J., Vecchio A.J., Malkowski M.G. (2016). Crystal structure of aspirin-acetylated human cyclooxygenase-2: Insight into the formation of products with reversed stereochemistry. Biochemistry.

[26] Cera E.D. (1995). Thermodynamic Theory of Site-Specific Binding Processes in Biological Macromolecules.

[27] Gohlke H., Klebe G. (2002). Approaches to the description and prediction of the binding affinity of small-molecule ligands to macromolecular receptors. Angew. Chem. Int. Ed..

[28] Sportoletti G., Testi V. (1985). Amino-Salicylic Acid Derivatives and Pharmaceutical Compositions.

[29] Serhan C.N. (2007). Resolution phase of inflammation: Novel endogenous anti-inflammatory and proresolving lipid mediators and pathways. Annu. Rev Immunol..

[30] Cherezov V., Rosenbaum D.M., Hanson M.A., Rasmussen S.G., Thian F.S., Kobilka T.S., Choi H.J., Kuhn P., Weis W.I., Kobilka B.K. (2007). High-resolution crystal structure of an engineered human beta2-adrenergic G protein-coupled receptor. Science.

[31] Gurevich V.V., Gurevich E.V. (2017). Molecular Mechanisms of GPCR Signaling:A Structural Perspective. Int. J. Mol. Sci..

[32] Filmore D. (2004). It’s a GPCR world. Mod. Drug Discov..

[33] Overington J.P., Al-Lazikani B., Hopkins A.L. (2006). How many drug targets are there?. Nat. Rev. Drug Discov..

[34] Hauser A.S., Attwood M.M., Rask-Andersen M., Schiöth H.B., Gloriam D.E. (2017). Trends in GPCR drug discovery: New agents, targets and indications. Nat. Rev. Drug Discov..

[35] Tang T., Gong T., Jiang W., Zhou R. (2018). GPCRs in NLRP3 Inflammasome Activation, Regulation, and Therapeutics. Trends Pharmacol. Sci..

[36] Bermudez M., Nguyen T.N., Omieczynski C., Wolber G. (2019). Strategies for the discovery of biased GPCR ligands. Drug Discov. Today.

[37] Muratspahić E., Freissmuth M., Gruber C.W. (2019). Nature-Derived Peptides: A Growing Niche for GPCR Ligand Discovery. Trends Pharmacol. Sci..

[38] Ashok S.R., Shivananda M.K., Manikandan A., Chandrasekaran R. (2019). Discovery and synthesis of 2-amino-1-methyl-1H-imidazol-4(5H)-ones as GPCR ligands; an approach to develop breast cancer drugs via GPCR associated PAR1 and PI3Kinase inhibition mechanism. Bioorg. Chem..

[39] Bantscheff M., Scholten A., Heck A.J.R. (2009). Revealing promiscuous drug-target interactions by chemical proteomics. Drug Discov. Today.

[40] Hu Y., Gupta-Ostermann D., Bajoratha J. (2014). Exploring Compound Promiscuity Patterns and Multi-Target Activity Spaces. Comput. Struct. Biotechnol. J..

[41] Sun L., Ye R.D. (2012). Role of G protein-coupled receptors in inflammation. Acta Pharmacol. Sin..

[42] Vane J.R. (1971). Inhibition of Prostaglandin Synthesis as a Mechanism of Action for Aspirin-like Drugs. Nat. New Biol..

[43] Vane J.R., Botting R.M. (1997). Mechanism of action of aspirin-like drugs. Semin. Arthritis Rheum..

[44] Ortiz M., Granados S.V., Castañeda H.G. (2006). Possible Activation of Inward Rectifier-and G Protein-Coupled K^+ Channels in the Antinociception Induced by Non-steroidal Anti-inflammatory Drugs. Proc. West. Pharmacol..

[45] Wulff H., Palle C. (2015). Recent developments in ion channel pharmacology. Channels.

[46] Gfeller D., Michielin O., Zoete V. (2013). Shaping the interaction landscape of bioactive molecules. Bioinformatics.

[47] Wang L., Ma C., Wipf P., Liu H., Su W., Xie X.-Q. (2013). TargetHunter: An in silico target identification tool for predicting therapeutic potential of small organic molecules based on chemogenomic database. AAPS J..

[48] Ramirez G., Coletto L., Sciorati C., Bozzolo E., Manunta P., Rovere-Querini P., Manfredi A. (2018). Ion Channels and Transporters in Inflammation: Special Focus on TRP Channels and TRPC6. Cells.

[49] Roberts M.S. (2007). Dermal Absorption and Toxicity Assessment.

[50] Pratt W.B., Pratt W.B., Taylor P. (1990). The entry, distribution, and elimination of drugs. Principles of Drug Action: The Basis of Pharmacology.

[51] Ajay A., Bemis G.W., Murcko M.A. (1999). Designing libraries with CNS activity. J. Med. Chem..

[52] Ma X.L., Chen C., Yang J. (2005). Predictive model of blood-brain barrier penetration of organic compounds. Acta Pharmacol. Sin..

[53] Geneve J.E.A.N., Hayat-Bonan B., Labbe G., Degott C., Letteron P., Freneaux E., Pessayre D. (1987). Inhibition of mitochondrial beta-oxidation of fatty acids by pirprofen. Role in microvesicular steatosis due to this nonsteroidal anti-inflammatory drug. J. Pharmacol. Exp. Ther..

[54] Dahl S.L., Ward J.R. (1982). Pharmacology, clinical efficacy, and adverse effects of the nonsteroidal anti-inflammatory agent benoxaprofen. Pharmacother. J. Hum. Pharmacol. Drug Ther..

[55] Zimmerman H.J. (1999). Hepatotoxicity: The Adverse Effects of Drugs and Other Chemicals on the Liver.

[56] Li C., Grillo M.P., Benet L.Z. (2003). In vivo mechanistic studies on the metabolic activation of 2-phenylpropionic acid in rat. J. Pharmacol. Exp. Ther..

[57] Boelsterli U.A. (2003). Diclofenac-induced liver injury: A paradigm of idiosyncratic drug toxicity. Toxicol. Appl. Pharmacol..

[58] Dong J.Q., Liu J., Smith P.C. (2005). Role of benoxaprofen and flunox-aprofen acyl glucuronides in covalent binding to rat plasmaand liver proteins in vivo. Biochem. Pharmacol..

[59] Tsutsui T., Hayashi N., Maizumi H., Huff J., Barrett J.C. (1997). Benzene-,catechol-, hydroquinone- and phenol-induced cell transformation, gene mutations, chromosome aberrations, aneuploidy, sister chro-matid exchanges and unscheduled DNA synthesis in Syrian hamster embryo cells. Mutat. Res. Fundam. Mol. Mech. Mutagen..

[60] Bolton J.L., Trush M.A., Penning T.M., Dryhurst G., Monks T.J. (2000). Role of quinones in toxicology. Chem. Res. Toxicol..

[61] Rannug U., Holme J.A., Hongslo J.K., Šrám R.J. (1995). An evalution of the genetic toxicityof paracetamol. Mutat. Res. Fundam. Mol. Mech. Mutagen..

[62] Gujral J.S., Knight T.R., Farhood A., Bajt M.L., Jaeschke H. (2002). Mode of cell death after acetaminophen overdose in mice: Apoptosis or oncotic necrosis?. Toxicol. Sci..

[63] Calder I.C., Hart S.J., Smail M.C., Tange J.D. (1981). Hepatotoxicity of phenacetin and paracetamol in the Gunn rat. Pathology.

[64] Kalgutkar A.S., Gardner I., Obach R.S., Shaffer C.L., Callegari E., Henne K.R., O’Donnell J.P. (2005). A comprehensive listing of bioactivation pathways of organic functional groups. Curr. Drug Metab..

[65] Nelson S.D., Forte A.J., McMurtry R.J. (1978). Decreased toxicity of the N-methyl analogs of acetaminophen and phenacetin. Res. Commun. Chem. Pathol. Pharmacol..

[66] Bursulaya B.D., Totrov M., Abagyan R., Brooks C.L. (2003). Comparative study of several algorithms for flexible ligand docking. J. Comput. Aided. Mol. Des..

[67] Cole J.C., Murray C.W., Nissink J.W.M., Taylor R.D., Taylor R. (2005). Comparing protein–ligand docking programs is difficult. Proteins Struct. Funct. Bioinf..

[68] Hevener K.E., Zhao W., Ball D.M., Babaoglu K., Qi J., White S.W., Lee R.E. (2009). Validation of molecular docking programs for virtual screening against dihydropteroate synthase. J. Chem. Inf. Model..

[69] Kontoyianni M., McClellan L.M., Sokol G.S. (2004). Evaluation of Docking Performance:  Comparative Data on Docking Algorithms. J. Med. Chem..

[70] Nissink J.W.M., Murray C., Hartshorn M., Verdonk M.L., Cole J.C., Taylor R. (2002). A new test set for validating predictions of protein-ligand interaction. Proteins Struct. Funct. Bioinf..

[71] Barcellos M.P., Santos C.B.R., Federico L.B., Almeida P.F.D., Silva C.H.D.P., Taft C.A. (2019). Pharmacophore and structure-based drug design, molecular dynamics and admet/tox studies to design novel potential pad4 inhibitors. J. Biomol. Struct. Dyn..

[72] Borges R.S., Palheta I.C., Ota S.S.B., Morais R.B., Barros V.A., Ramos R.S., Silva R.C., Costa J.S., Silva C.H.T.P., Campos J.M. (2019). Toward of Safer Phenylbutazone Derivatives by Exploration of Toxicity Mechanism. Molecules.

[73] Costa J.S., Ramos R.S., Costa K.S.L., Brasil D.S.B., Silva C.H.T.P., Ferreira E.F.B., Borges R.S., Campos J.M., Macêdo W.J.C., Santos C.B.R. (2018). An in silico study of the antioxidant ability for two caffeine analogs using molecular docking and quantum chemical methods. Molecules.

[74] Ramos R.D.S., Costa J.D.S., Silva R.C., Costa G.V., Rodrigues A.B.L., Rabelo É.D.M., Santos C.B.R.D. (2019). Identification of Potential Inhibitors from Pyriproxyfen with Insecticidal Activity by Virtual Screening. Pharmaceuticals.

[75] Esser R., Berry C., Du Z., Dawson J., Fox A., Fujimoto R.A., Haston W., Kimble E.F., Koehler J., Peppard J. (2005). Preclinical Pharmacology of Lumiracoxib: A Novel Selective Inhibitor of Cyclooxygenase-2. Br. J. Pharmacol..

[76] Kurumbail R.G., Stevens A.M., Gierse J.K., McDonald J.J., Stegeman R.A., Pak J.Y., Gildehaus D., Miyashiro J.M., Penning T.D., Seibert K. (1996). Structural basis for selective inhibition of cyclooxygenase-2 by anti-inflammatory agents. Nature.

[77] Marnett L.J. (2000). Cyclooxygenase mechanisms. Curr. Opin. Chem. Biol..

[78] House H.O. (1972). Modern Organic Reactions.

[79] Patani G.A., LaVoie E.J. (1996). Bioisosterism: A Rational Approach in Drug Design. Chem. Rev..

[80] Biagi G., Giorgi I., Livi O., Nardi A., Calderone V., Martelli A., Martinotti E., LeRoy S.O. (2004). Synthesis and biological activity of novel substituted benzanilides as potassium channel activators. V. Eur. J. Med. Chem..

[81] Silverstein R.M., Bassler G.C., Morril T.C. (1994). Indentificação Espectrometria de Compostos Orgânicos.

[82] Lipinski C.A. (2000). Drug-like properties and the causes of poor solubility and poor permeability. J. Pharmacol. Toxicol. Methods.

[83] Lipinski C.A., Lombardo F., Dominy B.W., Feeney P.J. (2001). Experimental and computational approaches to estimatesolubility and permeability in drug discovery and developmentqsettings. Adv. Drug Deliv. Rev..

[84] Drwal M.N., Banerjee P., Dunkel M., Wettig M.R., Preissner R. (2014). ProTox: A web server for the in silico prediction of rodent oral toxicity. Nucleic Acids Res..

[85] Santos C.B.R., Ramos R.S., Ortiza B.L.S., Silva G.M., Giuliatti S., Navarrete J.L.A., Carvalho J.C.T. (2018). Oil from the fruits of Pterodon emarginatus Vog.: A traditional anti-inflammatory. Study combining in vivo and in silico. J. Ethnopharmacol..

[86] (2007). Derek for Windows.

[87] Trott O., Olson A.J. (2010). AutoDock Vina: Improving the speed and accuracy of docking with a new scoring function, efficient optimization and multithreading. J. Comput. Chem..

[88] Dallakyan S., Olson A.J. (2015). Small-molecule library screening by docking with PyRx. Methods Mol. Biol..

[89] BIOVIA Dassault Systèmes (2017). BIOVIA Discovery Studio Visualizer.

[90] Turner G.W., Tedesco E., Harris K.D., Johnston R.L., Kariuki B.M. (2000). Implementation of lamarckian concepts in a genetic algorithm for structure solution from powder diffraction data. Chem. Phys. Lett..

[91] Bittencourt J.A.H.M., Neto M.F.A., Lacerda P.S., Bittencourt R.C.V.S., Silva R.C., Lobato C.C., Silva L.B., Leite F.A., Zuliani J.P., Rosa J.M.C. (2019). In silico evaluation of ibuprofen and two benzoylpropionic acid derivatives with potential anti-inflammatory activity. Molecules.

[92] dos Santos K.L.B., Cruz J.N., Silva L.B., Ramos R.S., Neto M.F.A., Lobato C.C., Ota S.S.B., Leite F.H.A., Borges R.S., da Silva C.H.T.P. (2020). Identification of Novel Chemical Entities for Adenosine Receptor Type 2A Using Molecular Modeling Approaches. Molecules.

[93] Santos C.B.R., Santos K.L.B., Cruz J.N., Leite F.H.A., Borges R.S., Taft C.A., Campos J.M., Silva C.H.T.P. (2020). Molecular modeling approaches of selective adenosine receptor type 2A agonists as potential anti-inflammatory drugs. J. Biomol. Struct. Dyn..

[94] Leão R.P., Cruz J.V., da Costa G.V., Cruz J.N., Ferreira E.F.B., Silva R.C., de Lima L.R., Borges R.S., dos Santos G.B., Santos C.B.R. (2020). Identification of New Rofecoxib-Based Cyclooxygenase-2 Inhibitors: A Bioinformatics Approach. Pharmaceuticals.

[95] Araújo P.H.F., Ramos R.S., da Cruz J.N., Silva S.G., Ferreira E.F.B., de Lima L.R., Macêdo W.J.C., Espejo-Román J.M., Campos J.M., Santos C.B.R. (2020). Identification of Potential COX-2 Inhibitors for the Treatment of Inflammatory Diseases Using Molecular Modeling Approaches. Molecules.

[96] Cruz J.V., Giuliatti S., Alves L.B., Silva R.C., Ferreira E.F.B., Kimani N.M., Silva C.H.T.P., de Souza J.S.N., Espejo-Román J.M., Santos C.B.R. (2021). Identification of novel potential cyclooxygenase-2 inhibitors using ligand- and structure-based virtual screening approaches. J. Biomol. Struct. Dyn..

[97] Koster R., Anderson M., Ej D.B. (1959). Acetic acid for analgesic screening. Fed. Proc..

[98] Woolfe G., MacDonald A.D. (1944). The evaluation of the analgesic action of pethidine hydrochloride (demerol). J. Pharmacol. Exp. Ther..

